# A behaviorally anchored assessment tool for bedside teaching in the emergency department

**DOI:** 10.1002/aet2.10789

**Published:** 2022-08-11

**Authors:** Hamza Ijaz, Matthew Stull, Erin McDonough, Robbie Paulsen, Jeffrey Hill

**Affiliations:** ^1^ University of Cincinnati Cincinnati Ohio USA; ^2^ University Hospitals Cleveland Medical Center Cleveland Ohio USA

## Abstract

Evaluating a resident's development as a bedside educator in the emergency department (ED) is challenging. Teaching consults, where trainees are observed and assessed in their teaching skills, have been used to improve bedside teaching. Within emergency medicine, there are a few assessment tools to evaluate a clinician's bedside teaching, with the majority focusing on faculty. A user‐friendly assessment tool adapted to the ED that emphasizes behaviorally anchored, milestone‐based evaluations for residents has yet to be developed. We sought to develop such an assessment tool for evaluating residents' bedside teaching in the ED. Using a nominal‐group consensus‐building technique, we derived the bedside teaching assessment tool. The consensus‐building panel was composed of clinician‐educators with extensive experience in resident education. The teaching consult process consisted of the consultant, a faculty member with a focus in medical education, directly observing a resident's bedside teaching throughout their shift while filling out the evaluation form based on observed behaviors. A total of 35 consults were provided to 30 individual residents. The mean (±SD) scores for the 35 consults for the learning climate, content teaching, supervision, feedback and evaluation, and self‐assessment were 3.84 (±0.75), 3.56 (±0.58), 3.70 (±0.60), 3.64 (±0.77), and 3.92 (±0.45), respectively. The median scores for the above domains were 4, 3.5, 4, 3.5, and 4, respectively. The tool has acceptable internal consistency with a Cronbach's alpha of 0.723 (95% CI 0.469–0.839). Eleven of 13 (85%) residents who provided feedback agreed or strongly agreed that the quantitative feedback provided by the assessment tool was useful. Twelve of 13 (92%) residents found the consultation process to be unobtrusive to their clinical performance. In conclusion, this novel behaviorally anchored assessment tool for bedside teaching can serve as a useful adjunct to a teaching consult and provide useful feedback for the development of residents' bedside teaching skills.

## NEED FOR INNOVATION

Evaluating a resident's development as an educator in the emergency department (ED) is challenging. Within emergency medicine (EM), there are a few assessment tools that evaluate bedside teaching, with the majority focusing on faculty teaching behaviors. Specifically, there are no tools focused on evaluating a resident's progression as an educator. The Stanford Faculty Development Program (SFDP) questionnaire was developed to evaluate faculty bedside teaching but focused on the inpatient setting.^1^, ^2^ The “Faculty Shift Card” sought to address this shortcoming by creating a bedside teaching assessment tool specific for the ED but it also focused on the faculty's teaching skills.^3^ Given the absence of a behaviorally anchored bedside teaching assessment tool for residents, we sought to create such a tool to improve their bedside teaching skills.

Teaching consults, where trainees receive a formative assessment of their teaching skills, have been used previously to address the challenge of evaluating clinical teaching methods.^4^ Assessment tools used during a teaching consult aim to improve the fidelity of the consult process by providing the consultee objective areas of strengths and weaknesses. An easy‐to‐use assessment tool adapted for the ED that emphasizes behaviorally anchored, milestone‐based evaluations for residents is a missing piece in the teaching consultant's toolbox. We utilized aspects of the Kirkpatrick Model, specifically learning and reaction, to evaluate the assessment tool and our teaching consult process.^5^

## BACKGROUND

Bedside teaching has numerous benefits. Trainees can demonstrate their communication and examination skills while educators can exemplify the humane aspects of practicing medicine.^6^ Clinicians seeking a formative assessment of their bedside teaching must ask an observer to utilize one of several evaluation forms that cover common themes including learning climate, student learning, goal setting, evaluation, feedback, and promoting self‐directed learning.^1^, ^2^, ^7^, ^8^, ^9^ These tools mainly focus on assessing faculty, not residents. Furthermore, tools assessing specific behaviorally anchored milestones for bedside teaching can potentially distinguish performance more clearly than standard Likert‐type scales.^10^, ^11^

The most utilized validated assessment tool for bedside teaching is the SFDP questionnaire.^2^ Although commonly used, it was designed to evaluate clinical teachers longitudinally in the inpatient environment, thereby limiting its applicability to the ED.^12^ The faculty shift card is specific to the ED and serves to facilitate on‐shift bedside teaching feedback from resident evaluators for their faculty by employing a behaviorally anchored assessment tool.^3^ It does not, however, assess a resident's bedside teaching. Behaviorally anchored milestones to assess bedside teaching are important as they can identify specific behaviors that are more objective compared to Likert scales, which can vary between evaluators. Furthermore, developing an observation‐based assessment has the potential to result in lasting adoption of specific teaching behaviors.^13^

## OBJECTIVE OF INNOVATION

We sought to develop an easy‐to‐use, behaviorally‐anchored assessment form for evaluating a resident's bedside teaching in the ED.

## DEVELOPMENT PROCESS

### Content validity

Using a nominal‐group consensus‐building technique, the study authors derived the bedside teaching assessment tool.^14^ The consensus‐building panel comprised three EM faculty involved in residency leadership, one in clerkship leadership, and one chief resident. Panel members performed independent literature searches to identify characteristics of high‐performing bedside educators. During the first meeting, the panel focused on arriving at 100% consensus on domains of assessment (learning climate, content teaching, supervision, feedback and evaluation, self‐assessment). In subsequent meetings, observable behaviors corresponding to levels of performance were identified and agreed upon, if they reached 100% consensus.

### Response process validity

**FIGURE 1:**
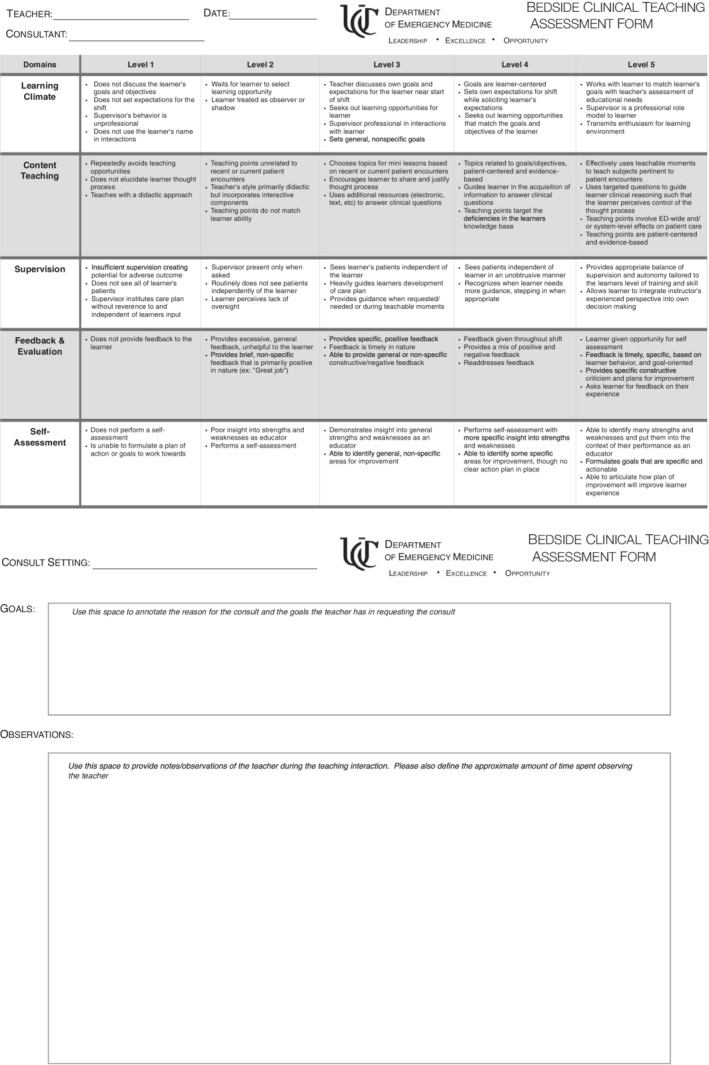
A behaviorally‐anchored assessment tool for evaluating resident bedside teaching in the ED.

The assessment form was piloted by several panel members on their initial consults. Feedback from the pilot testing process did not result in significant changes to the form or structure of the assessment. The result of the development process was an instrument with five domains of assessment, each with five levels of achievement with a subset of observable teaching behaviors to guide the evaluator (Figure 1).

## IMPLEMENTATION PHASE

The assessment tool is used as one component of a bedside teaching consult provided to residents who are on a bedside teaching elective, who are members of the residency's education leadership academy (ELA), or who voluntarily request a consult as a PGY‐4, all of whom had received a baseline level of bedside teaching training. The consultation process consisted of a preconsult meeting, direct observation, and a postconsult debriefing. The preconsult meeting covered the goals and areas of focus for the observation. The direct observation included an approximately four‐hour period of observation in the ED where the assessment tool was filled out in real time by an EM faculty with experience in EM education leadership. The postconsult debriefing included quantitative and qualitative feedback from the assessment tool.

## OUTCOMES/EVALUATION

Since 2014, a total of 35 consults were provided to 30 residents. Of the 30 residents, five residents requested and received two consults as part of a longitudinal evaluation. The mean (±SD) scores for the 35 consults for the learning climate, content teaching, supervision, feedback and evaluation, and self‐assessment were 3.84 (±0.75), 3.56 (±0.58), 3.70 (±0.60), 3.64 (±0.77), and 3.92 (±0.45), respectively. The median scores for the above categories were 4, 3.5, 4, 3.5, and 4, respectively. The assessment tool has acceptable internal consistency with Cronbach's alpha of 0.723 (95% CI 0.469–0.839). The sample size of the five residents' consultations as part of a longitudinal evaluation was too small to determine statistical significance.

Residents who participated in the consultation process provided feedback on the assessment tool through a postconsultation survey. Thirteen of 30 (43%) residents completed the postconsultation survey. Eleven of 13 (85%) respondents agreed or strongly agreed the quantitative feedback provided by the assessment tool was useful. All 13 respondents agreed or strongly agreed that the qualitative feedback provided through the consultation process and with the assessment tool was useful. Twelve of 13 (92%) respondents agreed or strongly agreed that the consultation process was unobtrusive to the performance of their clinical duties. Twelve of 13 (92%) respondents agreed that the consult was of solid or exceptional value to their growth and development as a bedside educator.

## REFLECTIVE DISCUSSION

The bedside teaching assessment tool developed as part of this project is unique in its applicability and usability in the ED to improve a resident's bedside teaching skills. This tool has a number of strengths and limitations.

The tool has evidence for content validity based on the development process. Based on a thorough literature search and a consensus‐building process that included both the chief resident and expert clinician educators, the tool incorporates many of the domains of previous tools but applies them directly to the ED. The assessment tool also has evidence for validity based on the consequences of testing. Postconsultation survey feedback illustrates that the consult process and use of the form was unobtrusive in real time and that both the quantitative and the qualitative information provided by the form were useful to growing as resident educators. However, feedback was provided by approximately 40% of residents, which must be taken into consideration when assessing the benefits of the consult process and the assessment tool.

There were some limitations to the instrument. We found that consultants utilized a relatively small section of the rubric. It is possible that this is due to the level of trainee involved in the consult process. Our residents receive training on bedside teaching throughout their residency curriculum and have medical students working with them on shift starting in their PGY‐2 year. It is possible that if applied to a set of trainees with less experience in bedside teaching, more of the rubric would be used. This finding, however, also raises the possibility that a more expansive delineation of observed behaviors at the higher end of the performance scale or a revision of the tool to eliminate the lower end of the performance scale may result in an assessment tool with greater utility for our particular trainees. It is also possible that residents are altering their teaching behaviors in the context of the consultation, resulting in higher levels of performance on the assessment.

We have learned several lessons over the years of using this tool. Broader application of teaching consults beyond the described population has been limited due to the time‐intensive nature of the consult process and a small group of trained faculty consultants. The assessment tool has been viewed by the faculty consultants as a useful adjunct to the consult process with the behavioral anchors serving as useful cues for the debriefing phase of the consult. Filling out the tool in real time during the observation phase allows the consultant to identify actionable behaviors that can be improved upon in future teaching opportunities. Repeated assessment over time would allow residents and faculty to track and demonstrate improvement in teaching skills.

In conclusion, this behaviorally anchored assessment tool for bedside teaching can serve as a adjunct to a teaching consult and provide residents feedback for the development of bedside teaching skills. This study highlights the processes undertaken in creating a bedside teaching assessment tool for residents. Further revision of this tool could lead to broader applicability for residents as well as for faculty.

## AUTHOR CONTRIBUTIONS

Hamza Ijaz—analysis and interpretation of data, drafting of the manuscript, and critical revision of the manuscript for important intellectual content. Matthew Stull—study concept and design and acquisition of the data. Erin McDonough—study concept and design; acquisition of the data; critical revision of the manuscript for important intellectual content; and administrative, technical, or material support. Robbie Paulsen—study concept and design; acquisition of the data; critical revision of the manuscript for important intellectual content; and administrative, technical, or material support. Jeffrey Hill—study concept and design, acquisition of the data, analysis and interpretation of the data, drafting of the manuscript, critical revision of the manuscript for important intellectual content, statistical expertise, and study supervision.

## CONFLICT OF INTEREST

The authors declare no potential conflict of interest.
